# Portraits of Living Physicians

**Published:** 1853-03

**Authors:** 


*Portraits of living Physicians.* — The zealous editor of the New Jersey Medical Reporter, proposes to introduce a new feature into medical Journalism in this country. He promises his patrons, occasionally, a portrait of some distinguished member of the profession, with a biographical sketch, selecting those who are natives of the state of New Jersey. Dr. George B. Wood, of Philadelphia, formerly of New Jersey, is to head the list.

We presume this effort to render the Reporter more attractive will be duly appreciated.

